# Efficient Avoidance of the Penalty Zone in Human Eye Movements

**DOI:** 10.1371/journal.pone.0167956

**Published:** 2016-12-08

**Authors:** Markku Kilpeläinen, Jan Theeuwes

**Affiliations:** 1 Institute of Behavioural Sciences, University of Helsinki, Helsinki, Finland; 2 Department of Cognitive Psychology, VU University Amsterdam, Amsterdam, The Netherlands; University of Muenster, GERMANY

## Abstract

People use eye movements extremely effectively to find objects of interest in a cluttered visual scene. Distracting, task-irrelevant attention capturing regions in the visual field should be avoided as they jeopardize the efficiency of search. In the current study, we used eye tracking to determine whether people are able to avoid making saccades to a predetermined visual area associated with a financial penalty, while making fast and accurate saccades towards stimuli placed near the penalty area. We found that in comparison to the same task without a penalty area, the introduction of a penalty area immediately affected eye movement behaviour: the proportion of saccades to the penalty area was immediately reduced. Also, saccadic latencies increased, but quite modestly, and mainly for saccades towards stimuli near the penalty area. We conclude that eye movement behaviour is under efficient cognitive control and thus quite flexible: it can immediately be adapted to changing environmental conditions to improve reward outcome.

## 1. Introduction

In everyday life we typically make around three to four saccadic eye movements every second [1,2]. Even though it may feel that these eye movements are made effortless without much, if any, cognitive control, research has demonstrated that eye movements are affected by a whole range of cognitive processes influencing basic eye movements parameters such as landing position, saccadic latency and saccade trajectories (reviewed in, [3–5]). Carpenter [6] argued that the oculomotor system provides ‘‘a microcosm of the brain itself”, allowing us to study at the lowest level the forces generated by eye muscles to move the eye, through the attentional mechanisms for locating relevant target objects, to the highest level at which decisions are made to move or not to move the eye. The current paper is concerned with the extent to which we are able to flexibly adjust the saccadic system when the circumstances require us to do so.

Response inhibition is a central index of the effectiveness and flexibility of cognitive control in various sensorimotor tasks [7,8]. Several paradigms have addressed the question whether it is possible to inhibit saccades when conditions require this. In the well-known oculomotor capture paradigm of Theeuwes and colleagues [9–11] participants had to avoid making a saccade to an irrelevant abrupt onset object that could appear at any location in the visual field. The results showed that in 20 to 30% of the trials, participants could not help making a saccade to the irrelevant distractor. Similarly, in the well-known antisaccade task, participants have to suppress making a prosaccade to a suddenly appearing visual stimulus on one side of fixation and instead make a saccade in the opposite direction [12,13]. In order to make such an antisaccade, one needs to suppress the saccade to the visual stimulus and actively and endogenously change the direction of the saccade in the opposite direction. These antisaccades typically have a much longer latency (about 250 to 350 ms) than saccades in the direction of the visual stimulus (about 150 to 250 ms). Typically, 10 to 20% of the saccades are erroneous in that they are made in the direction of the visual stimulus while they should have been made in the opposite direction [14].

The studies above all had in common that participants were required to stop making a saccade to an *object* appearing abruptly at an unpredictable location in the visual field. Eye movement programming, however, is profoundly location based (e.g., [15]). We thus asked here, whether participants can stop making saccades to a particular *well defined spatial area*, when the conditions change such that making a saccade to this area no longer results in reward but instead results in a monetary penalty. We also wanted to find out whether the possible costs of such a change in behaviour, in terms of response accuracy, time needed for learning and, especially, the slowing of responses are smaller in such a location avoidance task. In various response inhibition tasks, a delay-and-proceed type of strategy is often observed, where participants significantly delay all responses until the conflict between the need for inhibiting one response and carrying out another can be resolved [16,17]. Some previous studies have used reward and penalty contingencies to change eye movement behaviour. Schütz, Trommerhäuser and Gegenfurtner [18] showed that when saccades to particular regions were rewarded while saccades to other regions were penalized, it took participants about 100 trials to adjust saccade direction depending on the value of the region. Over the course of those 100 trials, participants strategically increased their saccadic latencies (with about 45 ms), suggesting that in this study participants had to learn to use a delay-and-proceed strategy to optimally harvest the rewards. Similarly, Blaukopf and DiGirolamo [19] introduced a reward and punishment scheme into an antisaccade task. They showed that reward and punishment schemes affected the programming of saccadic eye movements within a short time frame and in an online manner. However this adjustment came at a cost. When errors become more costly (because of the punishment), participants slowed their eye movement responses substantially and employed a more cautious strategy.

In the current study, we wanted to determine whether participants can avoid making saccades to a particular area of visual field even if a target appears within it. Participants had to make saccades to target stimuli, which were randomly presented at various distances from a well-defined penalty region, saccades to which involved a financial penalty. We also wanted to know whether participants would be able to benefit from knowing the constant location of the penalty region by applying an avoidance strategy more advanced than the general delay-and-proceed. To force participants to do so, a considerable time pressure (also involving financial incentives), was in place during the avoidance task.

## 2. Methods

### 2.1. Participants

Twelve university students (age 20–37, five female), all of whom reported normal visual acuity, participated in the study. The study adhered to the principles of the Declaration of Helsinki. The University of Helsinki ethical review board in humanities and social and behavioral sciences has reviewed the study and found that the study follows the ethical principles of research in the humanities and social and behavioral sciences issued by the Finnish Advisory Board on Research Integrity and is thus ethically acceptable (Decision 31/2013). The participants received a small compensation, partially dependent on their performance. The participants signed written consent, prior to the experiment.

### 2.2. Stimuli and apparatus

**Fig 1:**
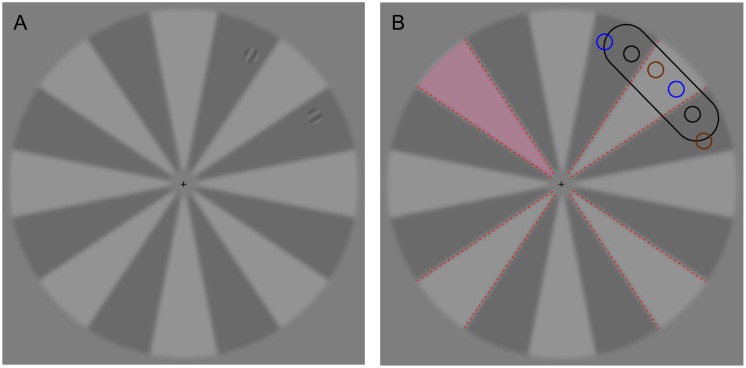
The stimulus setup. A) The targets, two grating stimuli, always 30° apart in polar angle, were presented at 7° eccentricity. B) The stimuli were presented randomly near the four diagonal sectors (red dashed lines), either symmetrically around the sectors (black circles) or displaced clockwise (orange circles) or counter clockwise (blue circles). The black oval shows the size of the hit area the saccade needed to land on in order to be considered accurate (see “Hit” in Table 1). The hit area moved with the target stimuli (here relative to the locations marked with black circles). The hit area was never shown to the participants. The penalty area could be one of the diagonal sectors, for example the top left one here marked with pink highlighting (see “Penalty sector” in Table 1). The pink highlighting was only shown to the participant for eight practice trials before the onset of the penalty phase.

The context stimulus was a circular shape with an inner radius of 1° and an outer radius of 8.2° of visual angle (see Fig 1), divided into 16 sectors (each 22.5° of polar angle). The luminance of the sectors alternated above and below mean luminance by 18.2 cd/m2 (14% contrast). The contrast polarity of the sectors was reversed between blocks. The global mean luminance of the context stimulus and the grey background areas was 130 cd/m2. The context stimulus was blurred slightly by convolving it with a 2-D Gaussian filter (SD 0.25°). The target stimuli were radially oriented, circular sine wave gratings (spatial frequency 2.6 cycles/deg, diameter 0.8°, Fig 1A). The Michelson contrast of the gratings was always 15% and the mean luminance of each grating was matched to the sector it was presented in. The two gratings always had the same phase, which reversed randomly.

The stimuli were created with Matlab 8 (Mathworks, Natick, MA, USA), running in a PC with an Nvidia Quadro K5000 (Nvidia, Santa Clara, CA, USA) graphics card, and presented with the Psychophysics Toolbox 3 [20] on a gamma corrected 22.5” VIEWPixx (VPixx Technologies Inc., Quebec, Canada) display with a 120 Hz refresh rate and 16-bit greyscale resolution (in the M16 mode). The viewable area of the display subtended 31 x 20°.

Eye movements were recorded with Eyelink 1000 (SR Research, Missisauga, Canada) video eye-tracker at 1000 Hz. Both pupil and corneal reflection were used for tracking. The eye-tracker was controlled by means of the Eyelink toolbox for Matlab [21]. The standard 9-point calibration was adjusted such that the points were 10% closer to the centre in order for them to better correspond with the used stimulus area. Mean error was required to be below 0.5° and maximum error below 1°. Calibration was repeated, if drift error approached 1° in a constant general direction or if the participants removed their head from the chin rest, but at least twice during the experiment.

### 2.3. Procedure

Each trial started with the appearance of the context stimulus and the central cross-hair (height 0.25°, see Fig 1A) and the participant indicating stable fixation with a space bar. If fixation was indeed stable, eye tracking and stimulus presentation started. Fixation cross was extinguished at a random delay of 600–800 ms after the accepted drift correction. Two target stimuli (Fig 1A) were then presented with a random delay of 0–150 ms relative to the disappearance of the fixation cross randomly at one of 12 possible location pairs, but always at 30° of polar angle from each other and at 7° eccentricity (see Fig 1B for example locations). The participants’ task was to move their gaze to either of the targets as quickly and accurately as possible. Each block included 48 trials, 12 around each diagonal sector. Of the twelve trials, in six the stimuli were placed symmetrically around the diagonal, in six they were displaced (see Fig 1B). There were altogether 16 blocks from which data was collected. Each participant had a personal penalty sector, the shape of which is indicated by the pink highlighting in Fig 1A, that stayed in constant location throughout the penalty phase. The different phases are described in 2.4.1–2.4.3. Each of the four diagonal sectors was used as the penalty sector for three participants. It must be emphasized that apart from a few practice trials, the penalty sector was physically completely identical to the other diagonal sectors. The only difference was that the participant knew that saccades to that sector could cause a penalty, and received a feedback if such a saccade occurred.

It was expected that the symmetrical arrangement (black circles in Fig 1B) would lead to a strong global effect (i.e., many averaging saccades, defined here as saccades that land closer to the midpoint between the two target stimuli than either of the stimuli), resulting in saccades to the penalty sector and that due to the strongly stimulus driven nature of averaging saccades [22–24] it would be difficult to avoid such saccades to the penalty sector. However, participants were equally effective in reducing the number of saccades to the penalty sector in all stimulus arrangements.

### 2.4 Rewards and penalties in different phases of the experiment

After each saccade, the participant received a feedback (a text on the screen) for the performance and the associated financial outcome (see Table 1). The system of financial outcomes was designed to motivate participants to make accurate saccades to target stimuli, but to avoid the penalty sector, all while under significant time pressure. For practical purposes, the financial outcomes were made such that the balance at the end of the experiment was relatively predictable (to the experimenter). Specifically, we did not want any participants to lose motivation during the experiment due to the balance approaching zero. Every participant started with a balance of 12 €. In the end of the experiment, the balance varied between 8–12 €.

**Table 1:** Feedback alternatives and financial outcomes.

Performance	Feedback	Outcome
Too slow	”The saccade was too slow!”	- 1.5 c
Penalty sector	“Saccade into the penalty sector!”	- 6 c
Miss	“The saccade missed the target.”	0 c
Hit	“The saccade hit the target spot on!”	+1.5 c

Table 1 presents the four possible performance types and their associated feedbacks (translated from Finnish) and financial outcomes. If two performance types apply to a saccade, the outcome higher on the list applies. Most importantly, if a saccade was too slow, the feedback and financial outcome concerned the slowness, regardless of where the saccade landed (in target area, in penalty sector, or elsewhere).

After each block of 48 trials, an instruction was displayed stating the block’s median latency and that saccades needed to be faster than that in the next block to be eligible for a reward. The balance was also displayed after each block. In the first block, in which a personal median could not be calculated, only saccades with latencies longer than 500 ms were considered too slow (which occurred seldom).

### 2.4.1 The pre-penalty phase

After participant setup and initial eye tracker calibration, the task and financial outcomes were explained (without mention of the penalty sector and the associated penalty). The participant then practiced the task for 24 trials, which did not affect the balance. After that, there were four (48 trial) blocks where the speed and accuracy of saccades affected the balance (see “Too slow”, “Miss” and “Hit” in Table 1).

### 2.4.2 The penalty phase

After the four blocks of the pre-penalty phase, the participant’s personal penalty sector and the associated 6 cent penalty were introduced. The participant then practiced the task for eight trials (with no effect on the balance), with the penalty sector highlighted (see Fig 1B). In four of the practice trials the targets were presented near the penalty sector. The pink highlighting on top of the penalty sector was only presented to the participant for those eight practice trials, not in the actual penalty condition blocks. After the introduction of the penalty sector, there were eight (48 trial) blocks in which, in addition to speed and accuracy, saccades to the penalty sector affected the balance (see “Penalty sector” in Table 1).

### 2.4.3 The post-penalty phase

After the eight penalty blocks, the participant was informed that the penalty condition was no longer in effect and that the possible financial outcomes were the same as in the pre-penalty phase: only speed and accuracy would affect the balance in the four remaining blocks.

### 2.5. Data processing

Saccades with the following characteristics were included in further analyses: onset latencies between 80 and 600 ms, start point within 1° from fixation, endpoint between 1.5 and 9° eccentricity, angular direction of the saccade within 30° from the closest target. The criteria led to an exclusion of 6% of trials.

**Fig 2:**
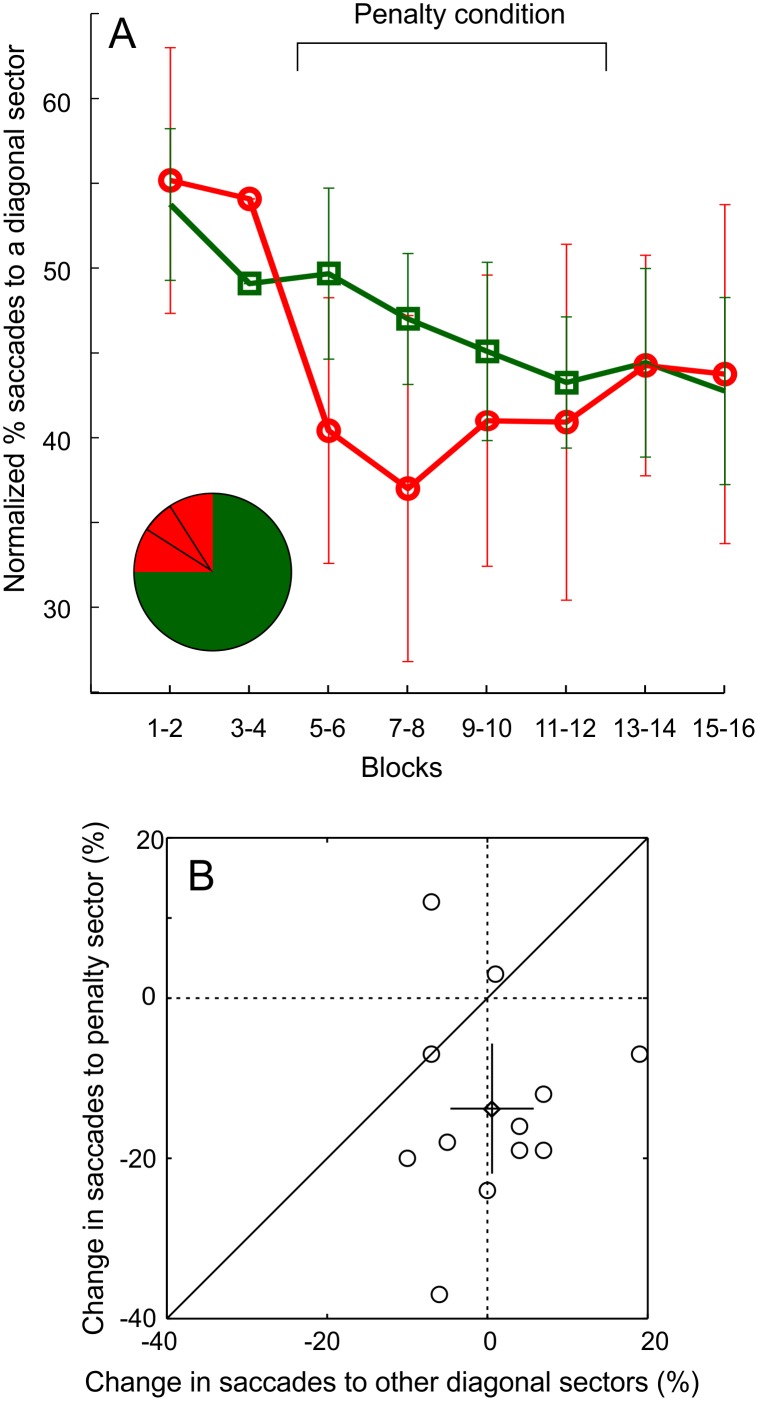
Participants instantly make fewer saccades to the penalty sector. A) The normalized proportion of saccades that landed within the penalty sector (red symbols, the smaller red segment in the inset), when the targets were presented in the quadrant that subsumed the penalty sector (red area in the inset) as a function of experimental blocks. For comparison, green symbols represent the proportion of saccades that landed on other diagonal sectors, when stimuli were presented in other quadrants. Error bars represent 95% CI. B) The instantaneous change (percentage units) in the proportion of saccades to the penalty sector against the change in the proportion of saccades to other diagonal sectors for 12 participants. The mean (and 95% CI) over subjects is indicated by the diamond marker (and the errorbars).

**Fig 3:**
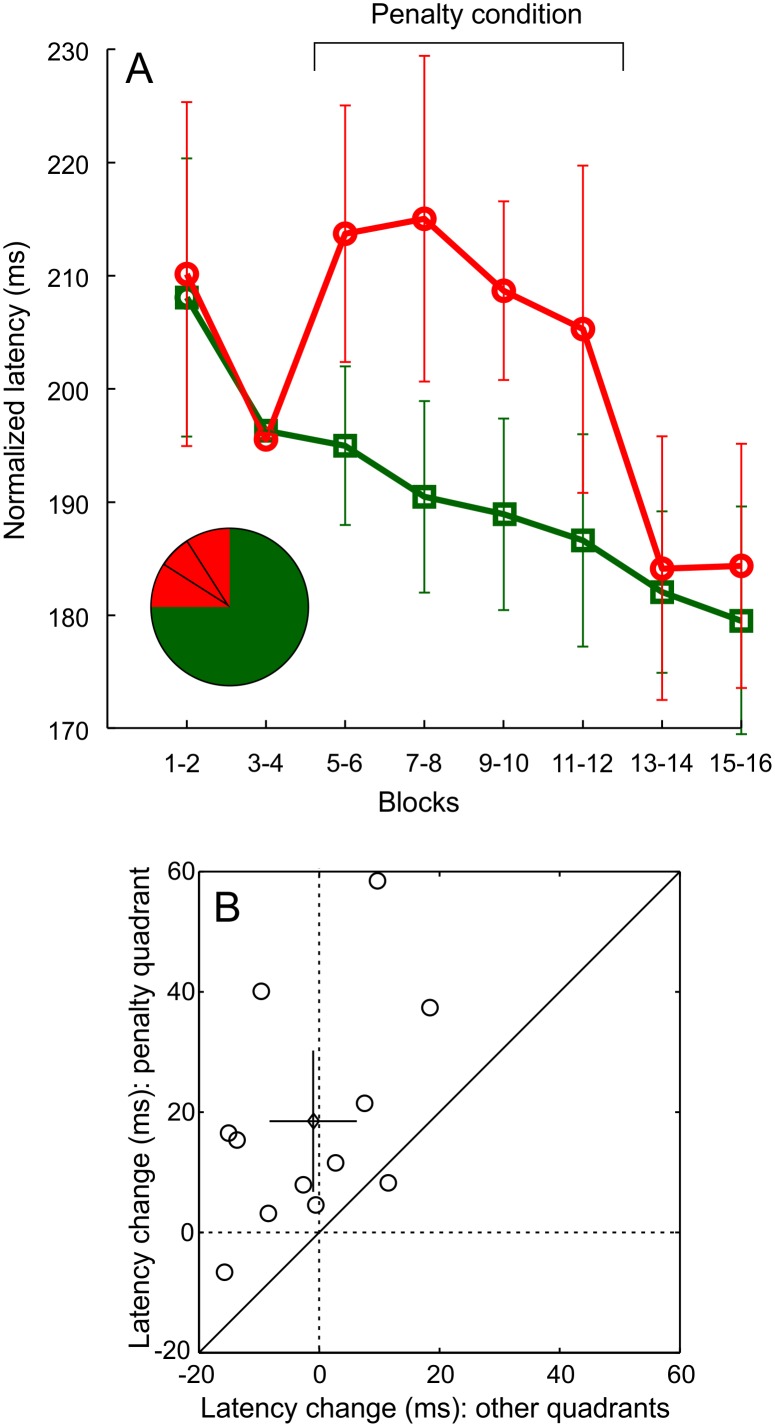
Participants slow down their saccades immediately after the onset of the penalty condition, but only for targets near the penalty sector. A) Normalized mean latency as a function of experimental blocks, separately for trials with targets in the quadrant with the penalty sector (red markers) and for trials with targets in other quadrants (green markers). Error bars represent 95% CI. B) The instantaneous change in mean latency for 12 participants: targets in the quadrant with the penalty sector vs. targets in other quadrants. The mean (and 95% CI) over subjects is indicated by the diamond marker (and the errorbars).

Data values used in figures and statistical analyses were averages calculated separately for each participant, experimental phase and other relevant factor (e.g., trials where targets were within the penalty quadrant and trials where targets were in other quadrants). For each phase, trials from two consecutive blocks were pooled together to reduce noise. Due to large inter-participant differences in overall saccade accuracy, and especially mean latency, data plotted in Figs 2 and 3 have been normalized by shifting the data of individual participants so that each participant’s value in blocks 3–4 equals the mean over participants. Blocks 3–4 were used rather than blocks 1–2, since in blocks 1–2 there was much less time pressure on the participants and results cannot be considered comparable to other phases of the experiment.

The rate of saccades to the penalty sector for each participant was calculated by dividing the number of saccades to the penalty sector (indicated by the smaller segment in the inset in Fig 2A) by the number of saccades to the entire quadrant (the red area in the inset). The rate of saccades to other diagonal sectors was calculated in the same way, but data for the three non-penalty quadrants was pooled together.

## 3. Results

### 3.1. Participants were able to reduce the number of saccades to the penalty sector

When one of the diagonal sectors of the background stimulus was revealed as the penalty sector, participants clearly reduced the proportion of saccades towards the penalty sector. The reduction was instantaneous for trials in which the two stimuli were presented in the quadrant that subsumed the penalty sector (the penalty quadrant, red symbols in Fig 2A). There was also a decrease in the number of saccades to other diagonal sectors (when stimuli were presented near or within them), but it was much more gradual (green symbols in Fig 2A). In a repeated measures ANOVA with the proportion of saccades landing in the penalty sector (or another diagonal sector) as a dependent variable, the phase of the experiment (blocks 1–2 vs 3–4 etc.) had a significant main effect (F(7,77) = 5.7, p < 0.01), but target positioning (targets in the penalty quadrant vs. other quadrants) did not (F(1,11) = 0.77, p > 0.05). Most importantly, however, the interaction effect of phase and target positioning (F(7,77) = 2.2, p < 0.01) was significant. Fig 2B shows that when proceeding from the last two blocks before the onset of the penalty condition to the first two blocks of the penalty condition, the proportion of saccades to the penalty sector decreases in 10/12 participants (data points below the dashed line). Further, it can be seen that at that point, participants initially avoided the penalty sector much more often than other diagonal sectors (data points below the diagonal). Whereas there was no change in the number of saccades to the diagonal sector in other quadrants, saccades to the penalty sector decreased on average by 14% units (t(11) = 3.4, p<0.01). We divided saccade latencies for each participant, phase (blocks 1–2 vs 3–4 etc.) and target position into four groups with an equal number of cases. In contrast to many earlier studies that suggest only slower saccades to be under top-down control [11,25–27], neither the main effect of latency group (F(3,33) = 1, p > 0.1) nor the interaction effects (latency group x positioning: F(3,33) = 0.3, p > 0.1; latency group x phase: F(21,231) = 1, p > 0.1; latency group x phase x positioning: F(21,231) = 0.3, p > 0.1) were significant. More specifically, the rate of saccades to the penalty sector was 44% in the fastest saccade bin (mean latency 170 ms) *after* the onset of the penalty condition, whereas it was about 53% in the slowest bin (mean latency 228 ms) *before* the onset of the penalty condition.

It appears from Fig 2A that the overall decrease in saccades to diagonal sectors, brought about by the penalty condition, is not reversed after the penalty condition is discontinued. Indeed, in a repeated measures ANOVA with the experimental phase (before vs. during vs. after penalty condition, main effect F(2,22) = 14.5, p < 0.01), the Bonferroni corrected pair-wise comparison yields a significant difference (p<0.01) between before and after penalty condition, but not between during and after penalty condition p>0.9).

It was expected that accurate saccades would become more difficult to make after the implementation of the penalty sector. There appeared to be a tendency of the proportion of accurate saccades to targets presented in the penalty quadrant to decrease during the penalty condition. However, in an ANOVA with hit rate as a dependent measure, and phase (blocks 1–2 vs 3–4 etc.) and target positioning as independent variables, neither the main effects (phase: F(7,77) = 0.7, p > 0.1; positioning: F(1,11) = 0.5, p > 0.1) nor the interaction (F(7,77) = 1.9, p > 0.1) were significant.

### 3.2. Participants selectively delayed saccades towards stimuli presented near the penalty sector

What did the participants change in their behaviour to allow the avoidance of the penalty sector? Quite expectedly, they slowed down their saccades. What is more remarkable, though, is that while participants substantially delayed saccades towards the quadrant of the penalty sector, saccades to other quadrants did not slow down, but instead gradually accelerated throughout the experiment. In a repeated measures ANOVA with mean latency as a dependent variable, the phase of the experiment (blocks 1–2 vs 3–4 etc.) had a significant main effect (F(7,77) = 10.7, p < 0.01) as did target position (targets in the penalty quadrant vs. other quadrants, F(1,11) = 5, p < 0.05) The interaction effect of phase and target position (F(7,77) = 7.3, p < 0.01) was also significant. Fig 3B shows that when going from the last two blocks before the onset of the penalty condition to the first two blocks of the penalty condition, latencies of saccades towards the penalty quadrant slowed down in 11/12 participants (data points below the dashed line). Further, it can be seen that at that point, participants slowed down their saccades towards the penalty quadrant more than towards other quadrants (data points above the diagonal). Whereas latencies of saccades towards other quadrants decreased by 1.3 ms, latencies of saccades towards the penalty quadrant increased on average by 18.2 ms (t(11) = 4, p<0.01). For trials with the stimuli in the penalty quadrant, there was no latency difference in the saccades that landed in the penalty sector or elsewhere (mean latencies during penalty phase: penalty sector 210 ms, elsewhere 208 ms, t(11) = 0.72, p>0.4.)

### 3.3 Participants improved the financial outcome

**Fig 4:**
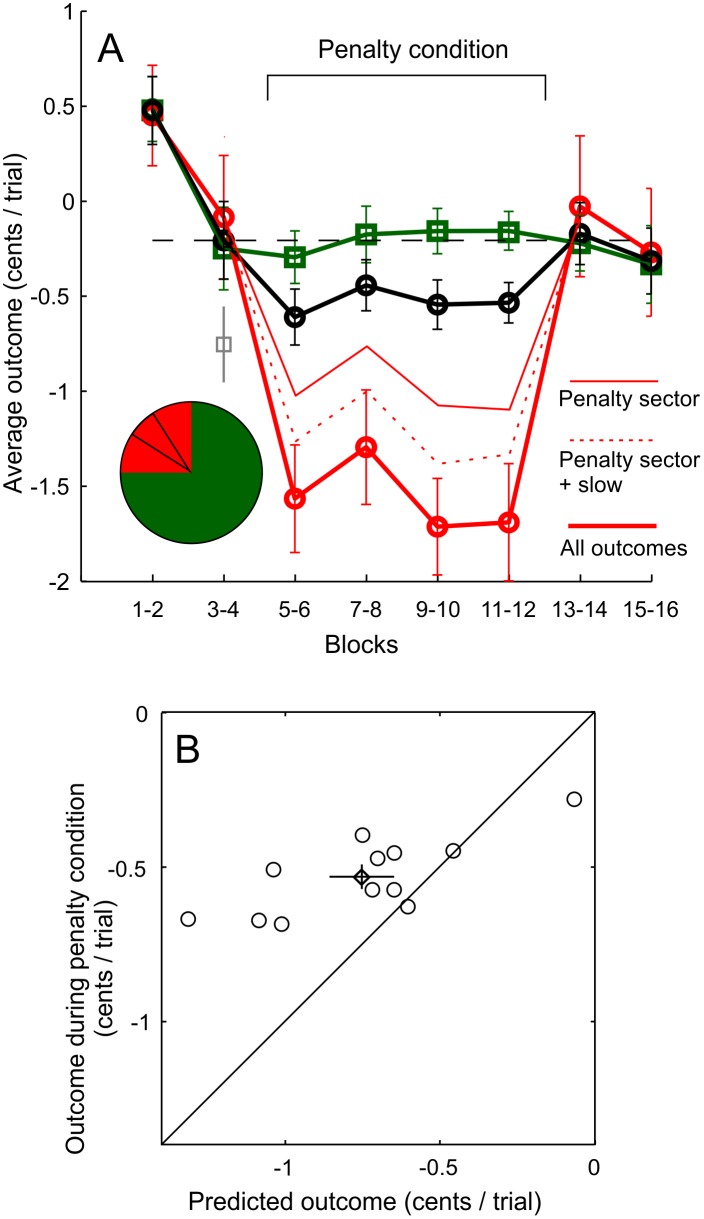
The financial outcome was worse during the penalty condition, but participants did improve their financial outcome by adapting their behavior. A) The financial outcome per trial as a function of experimental blocks, separately for trials where the targets were presented in the same quadrant with the penalty sector (red) and for trials with the targets in other quadrants (green) and for all trials (black). The thin red lines show individual contributions of different financial outcome types (see legend). The grey, solitary symbol at blocks 3–4 shows the prediction of the overall financial outcome if behaviour would not be adjusted at the start of the penalty condition. B) Mean outcome during the penalty condition vs. predicted outcome for 12 participants. The prediction is based on the participants’ behaviour during the last two blocks before the onset of the penalty condition. The mean (and 95% CI) over subjects is indicated by the diamond marker (and the errorbars).

The financial rewards and penalties were mainly designed to motivate participants to avoid the penalty sector, while keeping time pressure in effect. It is likely that participants tried to maximize their financial outcome. When the penalty condition was in effect, trials with the targets in the quadrant that subsumed the penalty sector were clearly quite costly to the participants (red symbols in Fig 4A). Saccades to the penalty sector had the largest effect on the financial outcome, as indicated by the area above the solid thin red line in Fig 4A, but saccades that were too slow (area between the solid and dashed thin red lines) and lack of rewards due to saccades that were slow or miss the target (area between dashed and thick red lines) also contributed to the financial outcome. In contrast, when stimuli were presented in other quadrants (3/4 of trials), participants performed roughly at the same level as before the start of the penalty condition (green symbols in Fig 4A), which also makes the overall financial loss more modest (black symbols in Fig 4A).

To determine whether the participants’ behavioural change was financially favourable, we compared the participant’s financial outcome during the penalty phase to what the outcome would have been if they continued behaving exactly as they did before the onset of the penalty condition, with no behavioural adjustment. According to this comparison, illustrated in Fig 4B, 10/12 participants adjusted their behaviour in a way that improved their financial outcome. Overall, the difference between the actual and predicted outcome is statistically significant. (t(11) = 3.1, p<0.01).

It can be observed from Fig 4B, that subjects differ much more in their predicted outcome than the actual outcome. Mainly two factors led to poor predicted outcome: high rate of averaging saccades (which lead to saccades to the penalty sector when the targets are on both sides of the penalty sector, e.g., the second lowest predicted outcome) or the inability to accelerate saccades (e.g., the lowest predicted outcome). In the former case, a reduction in the number of averaging saccades (with modest increas in saccade latencies) led to better than predicted actual outcome, in the latter case keeping saccade latencies relatively unchanged at the onset of the penalty phase was sufficient.

## 4. Discussion

The data indicate that participants were clearly able to avoid making saccades to a specific area of the visual field as soon as the area started carrying a financial penalty. This change in behaviour was accompanied by an increase in saccadic latencies. Remarkably, however, only saccades toward targets near the penalty area were delayed. The reduction in the number of saccades to the penalty area occurred immediately after the introduction of the penalty condition, indicating that little, if any learning was required to start using the avoidance strategy observed here.

### 4.1. Participants are able to avoid the penalty sector

In the current study, during the first 192 trials, participants were financially motivated to make fast and accurate saccades to target stimuli. After these initial 192 trials, one sector of the visual field became a penalty sector, implying that saccades to this area would result in a relatively large financial penalty. Importantly, incentives for speed and accuracy remained the same. The results showed that immediately after the introduction of the penalty sector, participants were able to significantly reduce the proportion of saccades towards the penalty sector. In a similar experiment, Schütz et al. [18] also found that participants successfully avoided a penalty incurring region. However in their study, it took almost 100 trials before participants learned to start avoiding this area. Also, in order to do so they slowed their saccades with about 45 ms, in close agreement with the latency difference of anti- and pro-saccades in an experiment by Ross, Viswanathan, Manoach, & Barton with comparable overall latencies and financial incentives [28]. Since participants in the studies by Ross et al. [28] and Schütz et al. [18] were free to use more time to deal with an increase in task complexity, both of the tasks can probably be successfully performed with the delay-and-proceed strategy, where all responses are slowed until a task appropriate response can be given [16]. That is not the case in the current study. Firstly, the latency increase observed here was clearly smaller, only about 18 ms. Some of the latency difference could have been caused by the strict time pressure that we imposed in the current study. Perhaps participants in the Studies by Ross et al. [28] and Schütz et al. [18] could have performed equally accurately, but somewhat faster, if forced to do so. The second difference between the current and earlier results, however, is more decisive. The latency increase in the current study concerned mostly trials where the targets were presented near the penalty region. In sum, it appears that the behavioural adaptation observed here was probably not achieved by means of the delay-and-proceed strategy, which involves a general delay of responses, but a more task tailored and specific strategy was needed.

Even though there is some resemblance between antisaccade tasks and the current task, it should be noted that in the current task, participants constantly knew the exact spatial area that is associated with a penalty, and this location did not change during the course of the experiment. It is feasible that these circumstances made it possible to inhibit an eye movement to the stationary penalty sector, while in antisaccade tasks the actual location which should be inhibited is not known in advance. Instead in the antisaccade task, participants need to suppress the urge to execute a saccade to a visual (onset) target. It is likely that active (constant or recurring) top-down inhibition of the (penalized) *spatial area* made it possible to suppress the saccade with such small time costs in the current study. Indeed, it has been claimed that within the saccade map (most likely in the superior colliculus) which is retinotopically organized, saccades to a particular area can be selectively supressed in a top-down fashion [11]. Research investigating selective attention has demonstrated that it is possible to selectively suppress the processing of information at a particular location [29,30]. For example, in Munneke et al. [29], cueing the location of an upcoming distractor reduced its distraction effect suggesting active top-down inhibitory mechanism. As attention and eye movements are strongly related [31,32], it is likely that attentionally suppressing a particular spatial area results in the suppression of the execution of a saccadic eye movement to that location. Such top-down suppression that is presumably already in effect at the start of a trial would also be consistent with our finding that the control of saccades was equally effective irrespective of saccade latency. In many earlier studies, in contrast, participants could only control the direction of relatively slower saccades [11,25,27].

In the current study, the penalty area was highlighted for 8 trials, and then remained stationary (but not highlighted) for the following 384 trials. It is likely that participants were able to avoid making saccades almost instantaneously without much learning because the area stayed (retinotopically) the same. This is unlike the experiment of Schütz et al. [18], in which the stimulus appeared randomly in one of four locations, and the relative positions of the penalty and reward regions of the stimulus flipped randomly across trials. This may explain why it took relatively long in Schütz et al.’s study (about 100 trials) to adjust the eye movement pattern and why a more costly delay-and-proceed strategy had to be implemented to avoid the penalty area.

We conclude that the successful avoidance of the penalty region observed here, which required little or no learning and caused only very modest slowing of saccades, could take place because participants were able to apply, possibly in a top-down manner, a constant inhibition to a specific spatial area in the visual field.

### 4.2 Behaviour after the penalty is removed

The effects of rewards often linger after rewards are no longer available [33] and this effect can be spatially localized with high precision [34]. In the present study, after the penalty condition was removed, participants accelerated the saccades towards targets near the penalty sector about as abruptly as they had decelerated them at the onset of the penalty phase (Fig 3A). The pattern of saccade rates to different areas is more complex, however. On one hand, there is no similar rebound in the rate of saccades to the penalty sector as there is in the latencies of saccades to the penalty quadrant. On the other hand, the rates of saccades to the penalty sector and to other diagonal sectors are equal after the end of the penalty condition, mainly because the latter gradually decreases through the penalty condition (Fig 2A). This decreasing trend of saccades to other diagonal sectors may simply be due to general learning or fatigue or some other factor that is unrelated to the penalty condition. However, it is important to point out the possibility that the initial selective avoidance of the penalty sector progresses into a general (automatic) tendency for avoiding all diagonal sectors, which always had the same luminance as the penalty sector. The spreading of inhibition could be mediated, for example, by perceptual grouping of the four diagonal sectors into a single shape (see Fig 1) or by simply avoiding all sectors with the same luminance as the penalty sector.

Whatever the reason for the observed pattern of saccade endpoints, the apparent discrepancy between the lack of change in saccade placement and the dramatic change in saccade latencies at the end of the penalty condition is interesting. It appears that during the last blocks before the end of the penalty condition, whenever targets are in the penalty quadrant, participants use an extra 15–20 ms on something that they can do equally well after the end of penalty condition, but without the extra latency. A behaviour pattern that has become automatic, possibly via one of the routes discussed above, seems one plausible cause for this.

### 4.3 Conclusions

In many real world situations it is beneficial to be able not to make saccades to distracting task-irrelevant regions in the visual field, as valuable time would be wasted attending to the irrelevant regions. The current study shows that people are able to avoid making saccades to particular areas in the visual field without much, if any, learning. Crucially, when doing so people selectively delay saccades only when targets are located nearby the penalty sector, and keep saccades to other regions of the visual field unaffected, indicating remarkably flexible cognitive control of oculomotor responses.

## References

[1] Andrews TJ, Coppola DM. Idiosyncratic characteristics of saccadic eye movements when viewing different visual environments. Vision Res. 1999 8;39(17):2947–53. 10492820 10.1016/s0042-6989(99)00019-x

[2] Rayner K. Eye movements in reading and information processing: 20 years of research. Psychol Bull. 1998;124(3):372–422. 9849112 10.1037/0033-2909.124.3.372

[3] Van Der Stigchel S, Meeter M, Theeuwes J. Eye movement trajectories and what they tell us. Neurosci Biobehav Rev. 2006;30(5):666–79. 10.1016/j.neubiorev.2005.12.001 16497377

[4] Schütz AC, Braun DI, Gegenfurtner KR. Eye movements and perception: A selective review. J Vis [Internet]. 2011 9 14 [cited 2012 Nov 9];11(5). Available from: http://www.journalofvision.org/content/11/5/9 10.1167/11.5.9 21917784

[5] Tatler BW, Hayhoe MM, Land MF, Ballard DH. Eye guidance in natural vision: reinterpreting salience. J Vis. 2011;11(5):5 10.1167/11.5.5 21622729 PMC3134223

[6] Carpenter RHS. The neural control of looking. Curr Biol. 2000 4 15;10(8):R291–3. 10801426 10.1016/s0960-9822(00)00430-9

[7] Aron AR. From Reactive to Proactive and Selective Control: Developing a Richer Model for Stopping Inappropriate Responses. Biol Psychiatry. 2011 6 15;69(12):e55–68. 10.1016/j.biopsych.2010.07.024 20932513 PMC3039712

[8] Verbruggen F, Logan GD. Response inhibition in the stop-signal paradigm. Trends Cogn Sci. 2008 11;12(11):418–24. 10.1016/j.tics.2008.07.005 18799345 PMC2709177

[9] Theeuwes J, Kramer AF, Hahn S, Irwin DE. Our Eyes do Not Always Go Where we Want Them to Go: Capture of the Eyes by New Objects. Psychol Sci. 1998 9 1;9(5):379–85.

[10] Theeuwes J, Kramer AF, Hahn S, Irwin DE, Zelinsky GJ. Influence of attentional capture on oculomotor control. J Exp Psychol Hum Percept Perform. 1999;25(6):1595 10641312 10.1037//0096-1523.25.6.1595

[11] Godijn R, Theeuwes J. Programming of endogenous and exogenous saccades: Evidence for a competitive integration model. J Exp Psychol Hum Percept Perform. 2002;28(5):1039–54. 12421054 10.1037//0096-1523.28.5.1039

[12] Hallett PE. Primary and secondary saccades to goals defined by instructions. Vision Res. 1978;18(10):1279–96. 726270 10.1016/0042-6989(78)90218-3

[13] Munoz DP, Everling S. Look away: the anti-saccade task and the voluntary control of eye movement. Nat Rev Neurosci. 2004 3;5(3):218–28. 10.1038/nrn1345 14976521

[14] Guitton D, Buchtel HA, Douglas RM. Frontal lobe lesions in man cause difficulties in suppressing reflexive glances and in generating goal-directed saccades. Exp Brain Res. 1985;58(3):455–472. 4007089 10.1007/BF00235863

[15] Schiller PH, Tehovnik EJ. Chapter 9 Look and see: how the brain moves your eyes about. In: Research B-P in B, editor. Elsevier; 2001 p. 127–42. (Vision: From Neurons to Cognition; vol. 134). 10.1016/s0079-6123(01)34010-4 11702539

[16] Aron AR, Behrens TE, Smith S, Frank MJ, Poldrack RA. Triangulating a Cognitive Control Network Using Diffusion-Weighted Magnetic Resonance Imaging (MRI) and Functional MRI. J Neurosci. 2007 4 4;27(14):3743–52. 10.1523/JNEUROSCI.0519-07.2007 17409238 PMC6672420

[17] De Jong R, Coles MGH, Logan GD. Strategies and mechanisms in nonselective and selective inhibitory motor control. J Exp Psychol Hum Percept Perform. 1995;21(3):498–511. 7790830 10.1037//0096-1523.21.3.498

[18] Schütz AC, Trommershäuser J, Gegenfurtner KR. Dynamic integration of information about salience and value for saccadic eye movements. Proc Natl Acad Sci. 2012 5 8;109(19):7547–52. 10.1073/pnas.1115638109 22529390 PMC3358910

[19] Blaukopf CL, DiGirolamo GJ. Differential effects of reward and punishment on conscious and unconscious eye movements. Exp Brain Res. 2006 9 15;174(4):786–92. 10.1007/s00221-006-0685-2 16977447

[20] Kleiner M, Brainard D, Pelli D. What’s new in Psychtoolbox-3? In: Perception 36 ECVP Abstract Supplement. 2007.

[21] Cornelissen FW, Peters EM, Palmer J. The Eyelink Toolbox: Eye tracking with MATLAB and the Psychophysics Toolbox. Behav Res Methods Instrum Comput. 2002 11;34(4):613–7. 12564564 10.3758/bf03195489

[22] Deubel H, Wolf W, Hauske G. The Evaluation of the Oculomotor Error Signal. In: Johnson AG G and F, editor. Advances in Psychology [Internet]. North-Holland; 1984 [cited 2016 Apr 4]. p. 55–62. (Theoretical and Applied Aspects of Eye Movement ResearchSelected/Edited Proceedings of The Second European Conference on Eye Movements; vol. 22). http://www.sciencedirect.com/science/article/pii/S016641150861818X

[23] Kilpeläinen M, Olivers CNL, Theeuwes J. The eyes like their targets on a stable background. J Vis. 2013 5 10;13(6):5 10.1167/13.6.5 23667242

[24] Van der Stigchel S, Nijboer TCW. The global effect: what determines where the eyes land. J Eye Mov Res. 2011;4:1–13.21603125

[25] Ludwig CJH, Gilchrist ID. Stimulus-driven and goal-driven control over visual selection. J Exp Psychol Hum Percept Perform. 2002;28(4):902–12. 12190257

[26] Chou I, Sommer MA, Schiller PH. Express averaging saccades in monkeys. Vision Res. 1999 12;39(25):4200–16. 10755158 10.1016/s0042-6989(99)00133-9

[27] Ottes FP, Van Gisbergen JAM, Eggermont JJ. Latency dependence of colour-based target vs nontarget discrimination by the saccadic system. Vision Res. 1985;25(6):849–62. 4024483 10.1016/0042-6989(85)90193-2

[28] Ross M, Lanyon LJ, Viswanathan J, Manoach DS, Barton JJS. Human prosaccades and antisaccades under risk: effects of penalties and rewards on visual selection and the value of actions. Neuroscience. 2011 11 24;196:168–77. 10.1016/j.neuroscience.2011.08.006 21846493

[29] Munneke J, Van der Stigchel S, Theeuwes J. Cueing the location of a distractor: An inhibitory mechanism of spatial attention? Acta Psychol (Amst). 2008;129(1):101–107. 18589391 10.1016/j.actpsy.2008.05.004

[30] Ruff CC, Driver J. Attentional Preparation for a Lateralized Visual Distractor: Behavioral and fMRI Evidence. J Cogn Neurosci. 2006 4 1;18(4):522–38. 10.1162/jocn.2006.18.4.522 16768358

[31] Deubel H, Schneider WX. Saccade target selection and object recognition: Evidence for a common attentional mechanism. Vision Res. 1996 6;36(12):1827–37. 8759451 10.1016/0042-6989(95)00294-4

[32] Godijn R, Theeuwes J. Parallel allocation of attention prior to the execution of saccade sequences. J Exp Psychol Hum Percept Perform. 2003;29(5):882–96. 10.1037/0096-1523.29.5.882 14585012

[33] Bucker B, Silvis JD, Donk M, Theeuwes J. Reward modulates oculomotor competition between differently valued stimuli. Vision Res. 2015 3;108:103–12. 10.1016/j.visres.2015.01.020 25668776

[34] Chelazzi L, Eštočinová J, Calletti R, Gerfo EL, Sani I, Libera CD, et al Altering Spatial Priority Maps via Reward-Based Learning. J Neurosci. 2014 6 18;34(25):8594–604. 10.1523/JNEUROSCI.0277-14.2014 24948813 PMC6608215

